# Supplementary material for: Bayesian Decision Analysis: An Underutilized Tool in Veterinary Medicine
Source: Animals (Basel). 2022 Dec 4;12(23):3414. doi: 10.3390/ani12233414 (PMC9738643; doi:10.3390/ani12233414)
Supplement: Supplementary file 1 [file animals-12-03414-s001.zip › animals-2003530-supplementary.pdf]

animals

MDPI

Methods S1. Expanded Case Description (As Presented to Clinicians for Diagnostic Probability Estimates).

History

An approximately 1-year-old, castrated male ferret presented emergently with a 2-day history of lethargy and inappetence. The owners first noticed lethargy 2 days prior to presentation when all of the other ferrets (4) in the household ran out of the ferret cage, but the patient did not. Over the next 2 days, the patient continued to be lethargic and was not eating. The owners tried syringing him water as they felt he was dehydrated. One day prior to presentation, the patient developed diarrhea that was a normal color but had a raw egg-like consistency. At presentation, the owners mentioned that he was a "chewer," and in the last few days they had noticed some of the ferrets playing with Christmas tree ornaments. They were unsure if the patient had gotten into anything. The patient has had no previous health issues, and none of the other ferrets were affected.

Physical Examination

On physical exam, the patient was quiet, alert, and responsive. The patient appeared to be mildly painful without stimulus. Mucous membranes were pale but moist with a capillary refill time of < 2 seconds. Dehydration was estimated to be ≤ 5%. A fecal sample produced during the course of the exam was normal in color and texture. The patient weighed 1.28 kilograms with a body condition score of 4/9. The patient's heart rate was 320 beats per minute with no murmurs or arrhythmias auscultated. Respiratory rate was approximately 60 breaths per minute with no crackles or wheezes auscultated. Rectal temperature was measured at 105.1º F (40.6º C). Abdominal palpation revealed no mass effect and did not appear to exacerbate pain. Peripheral lymph node palpation revealed an asymmetrically enlarged left axillary lymph node, measuring approximately 1 cm in diameter. A full neurologic examination was not performed, but the patient was fully ambulatory. Fleas were noted on the patient's dorsum.

Diagnostic Imaging

Three-view whole body radiographs were obtained under inhalant restraint (Figure S1). The radiologist description of the radiographs was as follows:

"There is faint mineralization of the bronchial walls, most evident on the right lateral view. There is a 2 mm wide, rectangular, well-defined, soft tissue opacity at the level of the pleural fissure between the right cranial and middle lung lobes, that abruptly stops approximately 3 mm from the periphery and is confluent with the cardiac silhouette craniomedially. A thin pleural fissure line is present between the right middle and right caudal lung lobes. The cardiac silhouette, pulmonary parenchyma and pulmonary vasculature are within normal limits. The stomach is moderately distended with inhomogeneous soft tissue opaque material admixed with gas and multifocal, pinpoint mineral opacities. The small intestines are normal in diameter and homogeneously soft tissue opaque. Multiple segments of small intestine contain faint, pinpoint, mineral opacities similar to the stomach contents. The colon contains gas and partially formed fecal material. Caudal to the pelvis, superimposed with the rectum, on midline, is a small, 1 mm, irregularly shaped, well-defined mineral opacity. The popliteal lymph nodes are mildly subjectively enlarged, measuring approximately 7 mm x 4 mm bilaterally."

An abdominal ultrasound performed by an experienced user (though not a boarded radiologist or radiology resident) was suspicious for shadowing material in the stomach. The entire abdomen was scanned systematically, and no other abnormalities were noted. Unfortunately, no saved images were available.

Biochemistry & Hematology

Blood samples were drawn from the caudal vena cava while the patient was under inhalant restraint for radiography. Plasma biochemistry panel and complete blood count values are shown (Table S1).

Exploratory Laparotomy

Acting on a suspicion of a GI foreign body, this patient underwent an exploratory laparotomy. The stomach was noted to be full of food material with no foreign material identified. Portions of the gastric mucosa were noted to be erythematous, and a gastric biopsy was obtained. Intestinal color and thickness were thought to be normal, no enlarged mesenteric lymph nodes were identified, no pancreatic nodules were apparent visually, adrenal glands were a normal size and shape, the urinary bladder had a normal appearance as did the liver.

Cytology & Clinical Progression

A left axillary lymph node aspirate was performed per the owner's wishes. The cytologic interpretation noted that if the lymph node was enlarged the sample was consistent with reactive lymphoid hyperplasia.

The patient had an extremely prolonged recovery after surgery (several hours). The patient was receiving buprenorphine, subcutaneous fluids (venous access unable to be obtained), ceftiofur crystalline free acid, and maropitant. The following evening (~24 hours post-surgery), when the patient was sufficiently alert to receive oral medications, a metronidazole + amoxicillin-clavulanic acid + ranitidine protocol was initiated for treatment of suspected *Helicobacter* gastritis. Sucralfate was later added to this therapy. Two days post-surgery, the patient was still extremely lethargic, anorexic, and had a fever of 104.2º F (40.1º C). Despite the risk of exacerbating potential GI ulceration, a high dose of meloxicam (0.5 mg/kg) was administered to reduce fever. The next day, however, the patient's fever had increased to 105.1º F (40.6º C). Given the lack of response to therapy, the owner's elected to humanely euthanize the patient three days after initial presentation. Submission of gastric biopsy and necropsy were declined by the owner.

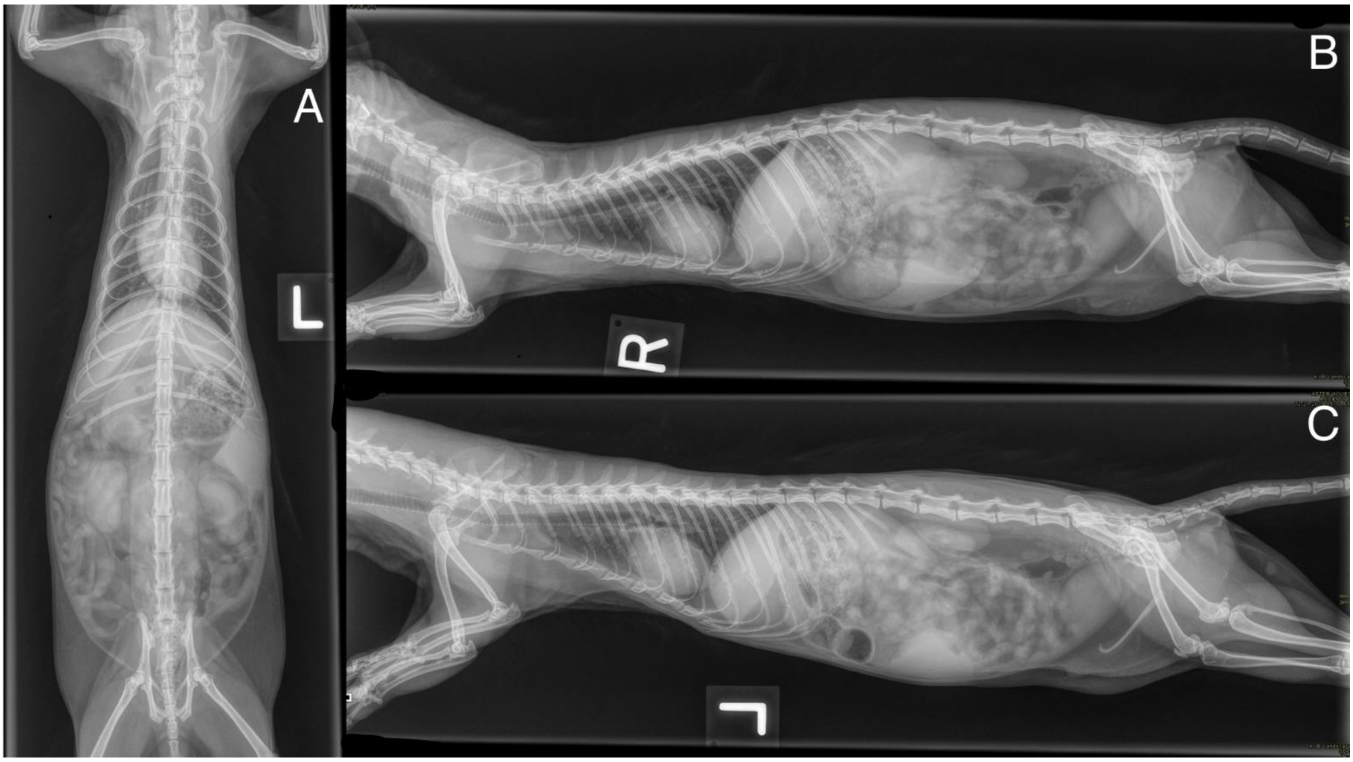

Figure S1. A) Whole body radiograph, ventrodorsal projection, of an approximately 1-year-old ferret presenting for two days of lethargy and inappetence and one day history of diarrhea; B) right lateral projection of the same patient; C) left lateral projection of the same patient.

Table S1. Biochemical and hematologic values for an approximately 1-year-old ferret presenting for two days of lethargy and inappetence and one day history of diarrhea.

**Plasma Biochemistry**			**Complete Blood Count**		
	Value	***Unit***		*Value*	*Unit*
Glucose	188	mg/dL	RBC	5.8	million/μL
AST	40	U/L	HGB	9.6	g/dL
ALT	120	U/L	HCT	29.3	%
ALP	24	U/L	MCV	50.6	fL
CK	159	U/L	MCHC	32.9	g/dL
TBIL	0.2	mg/dL	CHCM	31.4	g/dL
TP	5.1	g/dL	RDW	13.3	%
ALB	1.7	g/dL	PLT	169	k/μL
GLOB	3.4	g/dL	MPV	10.7	fL
BUN	21	mg/dL	PCT	0.18	%
CRE	0.5	mg/dL	PLT Comment	With many clumps	-
CA	7.9	mg/dL	Plasma Appearance	Slightly hemolyzed	-
PHOS	5.6	mg/dL	Protein	6.6	g/dL
NA	154	mmol/L	PCV	30	%
K	4.2	mmol/L	WBC	14.3	k/μL
CL	121	mmol/L	Seg. Neutrophils	9.4 (66)	k/μL (%)
Calculated Osmolality	312	mmol/L	Lymphocytes	3.4 (24)	k/μL (%)
			Monocytes	1.3 (9)	k/μL (%)
			Eosinophils	0.1 (1)	k/μL (%)
			Reticulocytes	29 (0.5)	k/μL (%)

Table S2. Individually estimated prior probabilities for each of 7 differential diagnoses and conditional probabilities of diagnostic findings given a known diagnosis. Posterior probabilities of differential diagnoses at time of surgery are shown. Each column shade corresponds to each outside clinician's estimates of prior, conditional, and posterior probabilities. For each diagnosis, a posterior probability was calculated using the following equation:.

$$\text{Posterior Probability} = \frac{\text{Prior Probability x Conditional Probability}_1 \text{ x Conditional Probability}_{n...}}{\text{Sum of All Diagnoses' Prior*Conditional Probabiliies}} \text{ x } 100$$

Diagnosis	Prior Probability (%)			Conditional Probability (%) Physical Exam			Conditional Probability (%) Radiography			Conditional Probability (%) Ultrasonography			Conditional Probability (%) CBC			Conditional Probability (%) Chemistry			Prior x Conditional Probabilities			Individual Posterior Probabilities (%)		
**Lymphoma**	10	8	10	5	60	10	25	40	10	5	20	10	20	75	10	5	75	10	625,000	2,160,000,000	1,000,000	7	92	0.003
GI Foreign Body	30	60	50	5	5	50	5	5	50	65	40	25	5	75	25	1	20	25	243,750	90,000,000	1,953,125,000	3	4	6
Systemic Coronaviral Infection	5	10	1	5	10	10	25	20	10	5	1	10	20	50	1	25	20	10	1,562,500	2,000,000	10,000	18	0.1	0.00003
Helicobacter Gastritis	20	2	10	10	5	10	10	50	25	5	40	25	20	20	50	25	50	50	5,000,000	20,000,000	156,250,000	59	0.9	0.5
Disseminated Idiopathic Myofasciitis	5	8	10	50	5	75	25	20	20	1	30	10	15	75	50	5	30	25	468,750	54,000,000	187,500,000	6	2	0.6
Bacterial Gastroenteritis	25	10	20	5	1	75	5	5	75	1	20	75	15	50	75	35	20	50	328,125	1,000,000	31,640,625,000	4	0.04	93
Unknown Toxicosis	5	2	1	20	1	10	5	50	10	20	50	10	5	75	1	5	50	1	250,000	18,750,000	1,000	3	0.8	0.000003
**SUM**																			8,478,125	2,345,750,000	33,938,511,000			

Table S3. Individually estimated, outside clinician-derived prior (posterior probabilities from Table S2 become prior probabilities due to a different reference time point) and conditional probabilities for differential diagnoses and diagnostic findings after all pre-surgical diagnostics in an approximately 1-year-old ferret presenting for two days of lethargy and inappetence and one day history of diarrhea. Posterior probabilities of differential diagnoses at time of euthanasia are also shown. Different column shades correspond to different outside clinician estimates of prior, conditional, and posterior probabilities. For each diagnosis, a posterior probability was calculated using the following equation:.

$$\text{Posterior Probability} = \frac{\text{Prior Probability x Conditional Probability}_1 \text{ x Conditional Probability}_{n...}}{\text{Sum of All Diagnoses' Prior x Conditional Probabiliies}} \text{ x } 100$$

Diagnosis	Prior Probability (%)			Conditional Probability (%) Exploratory Laparotomy			Conditional Probability (%) Lymph Node Cytology			Conditional Probability (%) Clinical Progression			Prior x Conditional Probabilities			Individual Posterior Probabilities (%)		
**Lymphoma**	7	92	0.003	20	50	1	60	40	5	20	20	5	176,926	184,163	0.07	85	99.1	0.000004
GI Foreign Body	3	4	6	1	10	25	5	10	5	20	10	5	288	384	3,597	0.1	0.00002	0.2

Systemic Coronaviral Infection	18	0.1	0.00003	1	10	10	20	10	5	20	10	10	7,372	9	0.015	4	0.0000004	0.0000008
Helicobacter Gastritis	59	0.9	0.5	30	75	75	3.4	10	5	3	50	5	18,046	639	863	9	0.00003	0.05
Disseminated Idiopathic Myofasciitis	6	2	0.6	15	20	1	3.3	10	5	15	75	75	4,105	460	207	2	0.00002	0.01
Bacterial Gastroenteritis	4	0.04	93	30	50	75	3.3	10	5	2	40	50	766	21	1,748,049	0.4	0.0000009	99.7
Unknown Toxicosis	3	0.8	0.000003	5	20	1	5	10	1	20	10	10	1,474	160	0.00003	1	0.000007	0.000000002
**SUM**													208,978	185,836	1,752,716			

"PROACTIVE" Mnemonic for Clinical Decision Analysis

- P – define the problem
  - What is the decision to be made?
- R – reframe from multiple perspectives
  - How are the problem and consequences viewed from the perspectives of veterinarian, client, public health, etc?
- O – focus on the objective
  - What is the main objective? Maximizing survival? Maximizing short-term quality of life?
- A – consider all relevant alternatives
  - Intervention A vs. intervention B vs. no intervention vs. euthanasia, etc.
- C – model the consequences and estimate the chances
  - Structure problem in a decision tree.
  - To calculate diagnosis probabilities, use Bayes' theorem.
    - The probability of a diagnosis given a set of clinical findings is proportional to the baseline probability of that diagnosis multiplied by the probability of those clinical findings given that diagnosis.
- T – identify the tradeoffs
  - What are the tradeoffs between each alternative? Expected survival vs. cost?
  - Assign utility values based on clinician's understanding of prognosis/cost and client values.
- I – integrate the evidence and values
  - From right to left, multiply utility values of outcomes by their probabilities.
  - For each chance node, sum the expected values to the right of the node.

Box S1. "PROACTIVE" mnemonic used to apply clinical decision analysis, adapted from *Decision Making in Health and Medicine: Integrating Evidence and Values, 2nd Edition* [6].
